# Improving AI-Based Clinical Decision Support Systems and Their Integration Into Care From the Perspective of Experts: Interview Study Among Different Stakeholders

**DOI:** 10.2196/69688

**Published:** 2025-07-07

**Authors:** Godwin Denk Giebel, Pascal Raszke, Hartmuth Nowak, Lars Palmowski, Michael Adamzik, Philipp Heinz, Marianne Tokic, Nina Timmesfeld, Frank Martin Brunkhorst, Jürgen Wasem, Nikola Blase

**Affiliations:** 1 Institute for Health Care Management and Research University of Duisburg-Essen Essen Germany; 2 Department of Anesthesiology, Intensive Care and Pain Therapy University Hospital Knappschaftskrankenhaus Bochum Bochum Germany; 3 Center for Artificial Intelligence, Medical Informatics and Data Science University Hospital Knappschaftskrankenhaus Bochum Bochum Germany; 4 Knappschaft Kliniken GmbH Recklinghausen Germany; 5 Department of Medical Informatics, Biometry and Epidemiology Ruhr University Bochum Bochum Germany; 6 German Sepsis Society Berlin Germany

**Keywords:** artificial intelligence, AI, clinical decision support systems, CDSS, decision-making, care, interviews

## Abstract

**Background:**

Artificial intelligence (AI)–based systems are receiving increasing attention in the health care sector. While the use of AI is well advanced in some medical applications, such as image recognition, it is still in its infancy in others, such as clinical decision support systems (CDSS). Examples of AI-based CDSS can be found in the context of sepsis prediction or antibiotic prescription. Scientific literature indicates that such systems can support physicians in their daily work and lead to improved patient outcomes. Nevertheless, there are various problems and barriers in this context that should be considered.

**Objective:**

This study aimed to identify opportunities to optimize AI-based CDSS and their integration into care from the perspective of experts.

**Methods:**

Semistructured web-based expert interviews were conducted. Experts representing the perspectives of patients; physicians; caregivers; developers; health insurance representatives; researchers (especially in law and IT); and experts in regulation, market admission and quality management or assurance, and ethics were included. The conversations were recorded and transcribed. Subsequently, a qualitative content analysis was performed. The different approaches to improvement were categorized into groups (“technology,” “data,” “users,” “studies,” “law,” and “general”). These also served as deductive codes. Inductive codes were determined within an internal project workshop.

**Results:**

In total, 13 individual and 2 double interviews were conducted with 17 experts. A total of 227 expert statements were included in the analysis. Suggestions were heterogeneous and concerned improvements: (1) in the systems themselves (eg, implementing comprehensive system training involving [future] users; using a comprehensive and high-quality database; considering usability, transparency, and customizability; preventing automation bias through control mechanisms or intelligent design; conducting studies to demonstrate the benefit of the system), (2) on the user side (eg, training [future] physicians could contribute to a more positive attitude and to greater awareness and questioning decision supports suggested by the system and ensuring that the use of the system does not lead to additional work), and (3) in the environment in which the systems are used (eg, increasing the digitalization of the health care system, especially in hospitals; providing transparent public communication about the benefits and risks of AI; providing research funding; clarifying open legal issues, eg, those related to liability; and standardizing and consolidating various approval processes).

**Conclusions:**

This study offers several possible strategies for improving AI-based CDSS and their integration into health care. These were found in the areas of “technology,” “data,” “users,” “studies,” “law,” and “general.” Systems, users, and the environment should be taken into account to ensure that the systems are used safely, effectively, and sustainably. Further studies should investigate both the effectiveness of strategies to improve AI-based CDSS and their integration into health care and the accuracy of their match to specific problems.

**International Registered Report Identifier (IRRID):**

RR2-10.2196/62704

## Introduction

### Background

The use of artificial intelligence (AI), especially machine learning, is becoming increasingly common in all areas of life. This is also evident in health care. In July 2024, the US Food and Drug Administration database included 882 AI- and machine learning–enabled devices from various medical fields such as radiology (n=671), cardiovascular medicine (n=90), neurology (n=32), hematology (n=17), gastroenterology and urology (n=13), anesthesiology (n=9), ophthalmology (n=9), clinical chemistry (n=8), general and plastic surgery (n=6), microbiology (n=6), pathology (n=6), or orthopedics (n=5) [1]. AI is believed to have tremendous potential to revolutionize patient care and outcomes [2,3]. This is especially because of its ability to support diagnosing, individualization of treatment plans, and clinical decision-making [2,4]. AI-based clinical decision support systems (CDSS) offer the potential to support physicians in their work and to optimize patient outcomes. For example, this is evident in the context of sepsis. In this context, such systems can support prediction, diagnosis, subphenotyping, prognosis, and clinical management [5]. This can lead to earlier treatment [6] and ultimately to shorter hospital stays [6,7] as well as reduced mortality [6-8].

Given these potential positive effects, it is questionable why AI-based CDSS have only been used in isolated cases in real care. One possible reason could be the barriers to establishing such systems: regulators must approve them. The systems must be integrated into electronic health record systems, and they must be standardized to the extent that similar products operate in a similar way. Clinicians must be trained to use them, they must be updated over time, and payment must be organized [4]. Furthermore, user perception is a fundamental criterion that decides acceptance [9].

The literature describes a further wide range of problems and barriers in the context of AI-based CDSS [10-12]. These relate to AI or CDSS and a combination of both, AI-based CDSS. While some of the problems relate to technical integration and operational use [10,11], others relate to the legal and ethical framework [12]. Regarding strategies for implementing AI-based CDSS in clinical practice, a study conducted by Peek et al [13] analyzed the literature and concluded that perspectives from groups other than health care professionals (eg, patients, carers, AI developers, health care managers, and leaders, and especially regulators and policy makers) should be investigated.

### Objective

To investigate the perceived problems and possible improvements involved in integrating AI-based CDSS to health care and to develop a user-oriented requirements profile for the systems, we established the research project “KI@work” [14]. It was founded by the German Federal Joint Committee (01VSF22050).

As part of the project, a scoping review (Raszke P, unpublished data, April 2025) was conducted to collect fundamental evidence. On the basis of the results, we conducted interviews with experts to further investigate the following questions: (1) What problems and barriers hinder the integration of AI-based CDSS into health care? (2) What approaches might be used to enhance the quality of AI-based CDSS and their integration into care? While the first question was covered in a previous article [15], this paper especially focuses on the second. Thus, this study aims to gather expert opinions on how AI-based CDSS and their integration into care can be optimized.

## Methods

### Overview

Expert interviews were conducted to identify approaches to solve perceived problems in the context of AI-based CDSS. We followed the standards of O’Brien et al [16] and the 32-item COREQ (Consolidated Criteria for Reporting Qualitative Research) checklist (Multimedia Appendix 1) [17] in preparing this manuscript. Therefore, transparency is guaranteed in all aspects of our study. The underlying interviews and corresponding transcripts, have already been examined for existing problems and barriers in the context of AI-based CDSS from the perspective of experts.

### Theoretical Framework

There is some evidence of problems and barriers, but as the field of AI-based CDSS is relatively new, little is known about how to optimize its use. Therefore, qualitative research in the form of expert interviews was conducted to approach the topic in an explorative way. The interviews were conducted with experts in the context of AI-based CDSS. The interviewees represented a wide range of different areas and professions. The interviews had 2 objectives, and the proceeding remained consistent: they started with open questions and topics and then addressed more specific questions. Within each topic, we first asked about different problems and barriers in the context of AI-based CDSS. Then, we asked about ways to improve care with AI-based CDSS.

### Ethical Considerations

Because patient views were collected through representatives and not from patients themselves, and no personal data were collected, the Ethics Committee of the Medical Faculty of the University of Duisburg-Essen confirmed that no application for ethics approval was required. Before the conversations, experts were informed via email about the topic and the proceeding. The email also included information about privacy and that the conversations would be recorded and transcribed for data analysis. Furthermore, the interviewees were informed that they could end the conversations on their part at any time without giving a reason and without experiencing any disadvantages. All experts provided consent to participate. The interviews were recorded and either transcribed automatically in Microsoft Teams (Microsoft) or manually for Zoom (Zoom Video Communications) interviews. Revision and pseudonymization were subsequently performed by research assistants and quality-checked, each by one of the moderators. After quality checking, the transcripts were sent to the respective experts for review to ensure the accuracy of the content. Three experts made use of this option, and their feedback was incorporated into the transcripts. The video recordings were then deleted. Therefore, it is not possible to assign individual statements to individual experts unless recalled by participants or moderators.

### Participant Selection and Preliminary Information

The question “Who are the relevant stakeholders in the context of AI-based CDSS?” was discussed during a project meeting to create a list of experts to contact. The list included representatives of patients; physicians; caregivers; developers; statutory health insurance funds; researchers (especially in law and informatics); and experts in regulation, market admission, quality management or assurance, and ethical issues. On the basis of this list, appropriate institutions were sought and contacted to find eligible interview partners. If a consortium member had a connection to one of the identified institutions, they helped establish contact with a potential interview partner (n=7). Five (71%) of the 7 people contacted participated in an interview. In 2 (29%) cases, the person originally contacted arranged an interview partner within their institution. The remaining 8 interviewees were recruited independently of personal networks. In all cases except one, it was ensured that the interviewers had no prior contact with any of the interviewees.

Possible participants were contacted directly via email by the consortium. After contacting the experts, none of them declined to take part in the study. Four experts did not respond to the request. Ultimately, all target groups were represented in the interviews.

### Setting and Conducting the Interviews

All interviews were conducted on the web via the conferencing platforms Microsoft Teams (Microsoft) or Zoom (Zoom Video Communications). Interviews included 1 or a maximum of 2 experts at a time. Circumstances or events which had an unintended influence on the conversations are not known. The interviews were moderated by 1 person and attended by at least 1 other person from a pool of 3 possible moderators (GDG, PR, and NB). The characteristics of the moderators are listed in Multimedia Appendix 2. Notable relationships did not exist between the researchers and the interviewees. There was no clear position for or against the use of AI-based CDSS in health care.

An interview guideline was developed (based on the preliminary results of a scoping review conducted before the interviews) by the team of the University of Duisburg-Essen and quality was assured within the consortium. The guideline included questions about both problems and barriers in the context of AI-based CDSS, as well as questions about possible improvements. It was slightly adapted with stakeholder-specific questions. The core version can be found in Multimedia Appendix 3. The interviews took place between June and August 2023.

### Data Analysis

A content-structuring qualitative analysis following the study by Kuckartz [18] was conducted using MAXQDA (VERBI Software GmbH) based on the final transcripts. The analysis aimed to conduct a content- and topic-oriented evaluation, as well as the development of main topics and subtopics. The analysis was based on deductive codes to create an overarching systematization. These included six categories in which approaches for optimization were suggested: (1) “technology,” (2) “data,” (3) “user,” (4) “studies,” (5) “law,” and (6) “general.” After deductive coding, relevant expert statements were concisely summarized (GDG) and subsequently discussed in a workshop (GDG, NB, and PR). The workshop aimed to develop finer-grained groups for a more differentiated perspective. Similar statements within the 6 overarching categories were grouped into subcategories (inductive codes), which were then used for further systematization. Finally, the expert statements were coded according to the inductive codes and systematized within a matrix using Excel (Microsoft Corporation; Multimedia Appendix 4). Data analysis was finished in October 2023.

## Results

### Overview

In total, 17 experts were interviewed in 13 individual and 2 double interviews. The professional backgrounds of the experts are provided in Table 1. The intrinsic motivation and participation of the experts were high.

**Table 1:** Conducted interviews.

Number	Stakeholder	Setting	Date
1	Caregiver representative	Individual interview	June 5, 2023
2	Representative of quality management	Individual interview	June 5, 2023
3	Researcher—AI^a^ in health care	Individual interview	June 6, 2023
4	Medical product consultant	Individual interview	June 16, 2023
5	Caregiver representative	Individual interview	June 27, 2023
6	Caregiver representative	Double interview	June 27, 2023
7	Representative of quality assurance	Double interview	July 4, 2023
8	Representative of an ethics committee	Individual interview	July 17, 2023
9	Researcher—Social and health law	Individual interview	July 19, 2023
10	Medical product consultant	Individual interview	July 27, 2023
11	Developer of AI-based CDSS^b^	Individual interview	July 27, 2023
12	Patient representative	Individual interview	July 31, 2023
13	Physician representative (Intensive care)	Individual interview	August 8, 2023
14	Developer of AI-based CDSS	Individual interview	August 11, 2023
15	Representative of health insurance fund	Individual interview	August 17, 2023

^a^AI: artificial intelligence.

^b^CDSS: clinical decision support systems.

**Figure 1:**
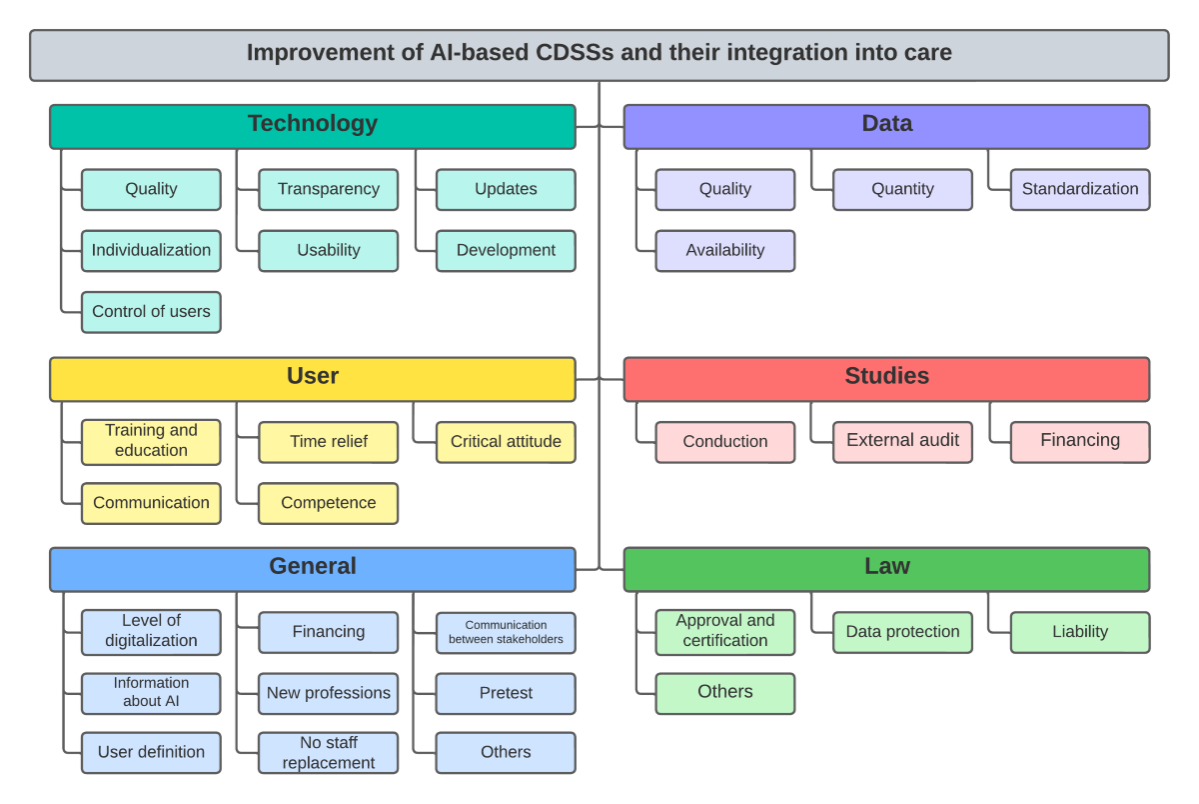
Identified improvement approaches matched with problem categories. AI: artificial intelligence; CDSS: clinical decision support systems.

A total of 227 expert statements included suggestions for improvement of AI-based CDSS and their integration into care. As described in the Methods section, these were categorized into 6 categories: “technology,” “data,” “user,” “studies,” “law,” and “general.” The systematization is presented in Figure 1. The complete and systematized list of summarized expert statements is presented in Multimedia Appendix 4.

### Technology

Optimization potential in the category of technology included “quality,” “transparency,” “updates,” “individualization,” “usability,” “development,” and “control of users.” Experts explained that the “quality” of AI-based CDSS is a fundamental factor affecting trust and acceptance over time. Therefore, requirements for high-quality systems should be considered. According to the experts, such requirements include that the systems be evidence-based and reliable and meet at least the current gold standard for value. Quality and validity assessments should be conducted regularly to maintain good quality over time. This should include checking the system against a validation dataset, sampling procedures, or test loops. The quality of systems could also reduce the burden of false alerts. Therefore, on the one hand, the systems should be able to identify false-positive and false-negative results. On the other hand, an appropriate balance between sensitivity and specificity might avoid unnecessary alerts as well as associated alert fatigue. Finally, incorrect recommendations can be avoided if systems clearly stick to evidence-based or, at least, consensus-based guidelines:

That means, the only reasonable method to increase acceptance, is to increase the quality of products and to gain trust in this way. That we currently have very low acceptance in some areas, maybe that’s a good thing and that it must increase over the next years is due to the innovative technology. A normal process.

Medical product consultant

“Transparency” can be guaranteed by avoiding black-box characters (incomprehensible AI). According to experts, AI-suggested decisions should be transparent, and systems should provide explanations. This could be achieved by identifying decisive parameters or using so-called heat maps to visualize data and give at least clues as to where the proposed decision stems from. Finally, it was mentioned that self-learning systems should stop evolving when they are no longer comprehensible:

So, the idea is actually that the decision that the AI gives me is justified by the AI in such way that I can, in a way that is so clear and comprehensible that I can perhaps also understand mistakes in the decision-making chain.

Caregiver representative

Experts stated that “updates” must be conducted to include new data and guidelines as well as to adapt the algorithm accordingly. Sensible implementation of user feedback should be considered during updates as well:

So, I demand diligence from the AI, but I also need the teacher somewhere, who must have a learning concept, the algorithm must always be continuously checked and adapted.

Representative of quality assurance

Experts with a more technical background saw “individualization” as a way of enhancing the quality of the systems. They explained that systems must be adapted to individual hospitals and that models from the United States should be optimized for local use in Germany. A physician representative, by contrast, stated that system standardization would be necessary to enable system use across locations:

I believe that you can’t do it preclinically. I have to talk to the hospitals, I have to set up the project in the hospital, in the care setting. Because [...] we have 1,200 ecosystems, if I’m only talking about acute hospitals now [...]. I have to involve the doctors in each hospital individually. I have to involve IT in every hospital.

Developer of AI-based CDSS

A further suggested approach lies in “usability.” User-friendliness should be considered to facilitate use and point users directly to relevant points. This includes the convenient use of AI-based CDSS for bedside conversations. User skills, as well as knowledge gaps, should be considered within system design. According to experts, a thorough “development phase” with a clearly determined question or target problem could contribute to the prevention of problems in the context of AI-based CDSS. The systems should be extensively and sufficiently developed and trained. False-positive and false-negative results must be checked during the training phase. The causes of identified errors must be eliminated. A balance between sensitivity, specificity, and overfitting (ie, training too specifically based on a fixed dataset) should be maintained. An interdisciplinary development, including experts and stakeholders from various fields (eg, future users, physicians, IT managers, and patient advocates) was desired. Involvement could include statements, tests of prototypes, participatory development, conceptual design, and open discourse. The development process should always be controlled by an independent process attendant:

I believe that it is crucial that, when you develop something AI-based like this, the people who will have to work with it later, who will have to use it, are involved as much as possible, right from the development phase.

Caregiver representative

Finally, “control of users” could improve the quality of AI-based CDSS and their integration into care. The intelligent design, implementation, and testing of risk control measures and their corresponding implementation in AI-based CDSS could prevent blind trust and automation bias. Furthermore, deliberately incorrectly proposed decisions by AI might be used to check the awareness of users:

Problems like this [such as automation bias], can be countered by designing and implementing intelligently enough, I would say.

Caregiver representative

### Data

A further starting point for improvement is the area of “data.” Approaches in this context concern the “quality,” “quantity,” “standardization,” and “availability” of data.

First, according to the experts, high data “quality” must be guaranteed. Therefore, the quality of the training data should be improved. This can be done through data management, reflective data synthesis, or reflective filling of incomplete datasets. The validity of the primary dataset should be made transparent:

So that would be a very important point for me, especially at the beginning of the study protocols, from our experience, also in terms of quality assurance, to be able to make a statement about the validity of the primary data pools.

Representative of quality assurance

“Quantity” included the enlargement of the database as well as its completeness. According to experts, both would contribute to better results. Structured and unstructured data should be included, and longitudinal data should be used in the analysis. No data should be excluded because of the age, origin, or sex of patients. Databases should be equally weighted, sufficiently represent women as well as minority individuals, and consider age and diseases:

And of course it is very important that we have equally weighted data sets, that we represent minorities and so on. But that’s a craft, it can be done. It has to happen, yes, but it’s not inherent in the technology, it’s simply a manual part of using the technology.

Researcher—AI in health care

A further approach was seen in “standardization.” Experts asked for national-level data standardization and integration. Therefore, data could be merged and made usable. Generic data patterns should be predefined:

[...] yes, I like, this generic character of data sets. This is, I rather think, the field, you have to create generic data patterns that hospitals have to adhere to in order to achieve accuracy.

Developer of AI-based CDSS

Finally, the “availability” of data could contribute to improving the systems and their integration into care. Experts suggested the creation of a national or European data pool, as well as networks, to obtain sufficiently large datasets. Data should also be accessible for the private sector to develop AI-based CDSS. Analogous data from hospitals should be made available using machine reading, such as optical character recognition, of PDF documents such as scans or photos. During the digitization process, experts should be involved in reading out subject-specific documents:

And that means we should take the EU Commission’s proposal with the draft regulation on the European Health Data Space seriously so that private sector companies that develop AI-based CDSS can also access such data. Because every year we have 550 million outpatient data records and 15 million inpatient data records that are not accessible for this development or not easily accessible.

Medical product consultant

### User

Many of the identified improvements are user-oriented. These include “training/education,” “time relief,” “critical attitude,” “communication,” and “competence.”

First, “training/education” might lead to more openness to AI-based CDSS. Therefore, information on AI should be a part of both medical training and further staff training. Staff training should include educational courses or workshops on the general topic of AI, its threats (eg, errors in its use, especially automation bias), its limitations, and information on its impact on existing care pathways. Courses should provide the opportunity to try out AI-based CDSS. Their application under stress should be exercised. In contrast to most of the other respondents, a developer stated that only those who modify the systems needed training but not those who just use the systems. A physician representative stated that trainings and courses should be nonmandatory. Experts pronounced that, within training, it should be clarified that AI will not replace humans:

And I think what would help to increase that acceptance would be just regular training. If you could also show examples of what a specific case might look like.

Caregiver representative

And I believe, if there are no training courses or no sensitization, then there is a risk that such recommendations for action will simply be adopted and that would be disastrous.

Developer of AI-based CDSS

Experts saw “time relief” of physicians and caregiving professionals as a solution for missing acceptance. They stated that the temporal valences of users should be considered and that AI should not lead to major additional work. Rather, it should make day-to-day work easier, for example, by supporting documentation. Furthermore, it was explained that clinicians should be relieved for scientific tasks in the context of AI and use their competence to assess complex cases:

[...] that you have a mixture of easier and more difficult cases, and AI will tend to mean that the easy cases can be checked off very quickly by the AI or with the help of the AI, and that only the very difficult cases will actually end up with the doctors. And maybe there needs to be some relief here. You have to say that somehow you have to take more breaks [...] because looking at the screen all day and then only having to differentiate between the worst cases is not easy.

Representative of an ethics committee

A further improvement approach was seen in the “critical attitude” of users. It was stated that decisions suggested by AI must always be scrutinized, validated, and checked for plausibility. There should be no blind reliance on AI. One expert explained that regardless of whether recommendations are made by AI or humans, they should always be questioned. A critical attitude was described as relevant to both positive and negative findings. Thus, even supposed false alarms should be taken seriously. One stakeholder wished for mutual skepticism between AI and users:

This means that the suggestions or recommendations for action of an AI-based CDSS must be evaluated by the doctor in the same way as a laboratory value or another parameter or a pathological ECG. It must be critically scrutinized; it must be validated [...].

Medical product consultant

In addition, an opposing view was taken in the context of “critical attitude”: users should gain greater trust in AI and, in some future cases, might also implement recommendations for action without checking them:

And at some point, we will receive recommendations for action that we can rely on 100% and then we can implement them without checking them. So, the answer to your question is: we encounter the problem by only implementing recommendations for action unchecked where we have a sufficiently high level of certainty that it will work due to the validation.

Medical product consultant

A last improvement approach concerning the user was seen in “competence.” According to experts, users need personal and professional competence to use and deal with decisions suggested by the AI, as well as to maintain decision-making authority and avoid dependence on AI. In particular, the implicit knowledge from experience should be maintained. Finally, interviewees also explained that trust increases with competence:

So, who decides and who does not, I think it has something to do with education, it has something to do with personal competence and of course it also has a lot to do with professional competence.

Caregiver representative

### Studies

Regarding “studies,” improvement approaches were named within 3 areas. These were “study conduction,” “external audit,” and “financing.”

“Study conduction” was seen as essential to prove evidence, to assess benefits, and to track side effects. Demonstrating success in studies would lead to increased public acceptance and trust. A general need for more valid (comparative) and prospective studies was pronounced. A “clean, best possible study design depending on the disease” [Representative of health insurance fund] was demanded. Orientation of studies on standards of scientific institutes and the involvement of experts was described as necessary:

We now need prospective randomized studies, one part gets standard care, one part gets AI and then we have to look and that has to be done on a relatively large number of patients across several sites so that we can see whether it is possible to detect organ dysfunction and sepsis in advance.

Physician representative—intensive care

In general, of course, the higher the quality of the study design - and a randomized controlled trial is of course the gold standard - the more reliable the results are.

Representative of health insurance fund

Experts stated that studies should also include an “external audit” to get a neutral view of the situation. Furthermore, involving patient organizations in studies can help increase acceptance among patients. Finally, in this context, an improvement approach was seen in the “financing”—specifically, the funding of studies. The federal or state governments or the European Union (EU) were seen as responsible for study funding:

We must now have this willingness to carry out these studies, [...] I mean we’re talking about a few million euros, [...]. That is now indicated, that is the next logical step and if we were to do that, we would be very far ahead, not just in Europe, but globally.

Physician representative—intensive care

### Law

“Approval/certification,” “data protection,” and “liability” were seen as possible improvement approaches within the category of “law.” Further suggestions within this category were aggregated under “others.”

“Approval/certification” and review of AI-based CDSS should be conducted regularly by independent bodies to increase acceptance. It was expressed that certifying bodies would need more staff with a background in science, technology, engineering, and mathematics. Approval of AI should not lead to major additional costs compared with conventional medical devices. Another approach expressed was a certification that authorizes humans to use AI:

I think independence is important. An established, independent institute and regular repetition, [...] and AI systems are also constantly changing, because they are - AI means a self-learning system, which means that you have to check them every now and then. So regularly, independently and in an understandable way.

Patient representative

Further opportunities were seen in the regulation of “data protection.” Data protection standards should always be appropriate, and data integration should be standardized nationwide. Users could benefit from already certified plug and play hardware solutions to avoid data protection issues. Whether researchers are allowed to use the collected data should be decided by the patients. Furthermore, experts stated that problems in the context of data protection are often caused by data protectors and not by data protection requirements and are thus avoidable:

Every data protection officer has a problem with personal data that goes off campus, and I absolutely understand that. But what I always like to ask is, has anyone ever asked the patients whether they could possibly care less? [...] There are clinics that do this in Germany, they use all their patients’ data for research purposes. It’s not forbidden, I’m just saying opt-in, opt-out.

Developer of AI-based CDSS

Regarding “liability,” experts have suggested that modes of action and responsibilities should be clarified. In this context, it was proposed that it should be legally anchored that diagnostic and therapeutic responsibility remains with the user, specifically the physician. Users should be informed about the legal framework. Otherwise, one expert explained that physicians should not be made liable for AI-proposed decisions that cannot be verified because of a black-box character of the systems:

And liability risks are also a major issue, and doctors naturally don’t want to be held liable for anything, for possible treatment errors or that they have overlooked something if they can’t actually assess the quality of this AI. And that’s why I would say that increasing acceptance is also possible through regulation.

Representative of an ethics committee

A further two improvement approaches were described. These were the obligations to report and publish errors that occur in the context of the systems in general and to report and track, especially, the causes of incorrect decision recommendations.

### General

Under the category “general,” various suggestions for improvement were summarized that did not match one of the other categories. These include “level of digitalization,” “financing,” “communication between stakeholders,” “information about AI,” “new professions,” “pretest,” “user definition,” “no staff replacement,” and “others.”

The “level of digitalization” should be expanded for IT infrastructure. There is a demand for high-performance computing to ensure fast, reliable, and failure-free operation of the systems. Digitization should be standardized to close gaps in the documentation:

And I remember that from before, when we had the paper curve, we really had a lot of gaps in the documentation as far as vital signs were concerned. So that’s why we should have uniform digitization, which would of course be the optimum.

Caregiver representative

Improvement in “financing” concerned 3 aspects. First, service providers should be motivated with incentives to use AI-based CDSS. Second, incentivization could lead service providers to collect data more conscientiously. Third, experts saw the federal states and the German social policy as responsible for participation in hospital financing and the financing of digitalization infrastructure of the health care system:

But on the whole, people act as if this is purely a private matter. [...] My personal opinion would be that German social policy should contribute to the financing of infrastructure within the digitalization of the healthcare system in particular [...].

Caregiver representative

“Communication between stakeholders” could be improved by using a common language between the professions. Experts suggested appointing a chief medical IT officer to build bridges between stakeholders and drive the integration of AI:

It’s not down to our doctors’ willingness to innovate, on the contrary, it’s a lot down to IT and a lot down to the data protection officer and a lack of communication within the hospital. My hospital is divided into two departments. On the one hand we have white, everything that is medicine and on the other hand it is a normal company. That’s why I think one of the most important positions in a company is the CMIO, the Chief Medical IT Officer, who has a view from both sides. And he can also build a very big bridge.

Developer of AI-based CDSS

Another much-discussed improvement category is “information about AI.” In this context, communication should include both the benefits and the limits of AI. According to experts, education and transparent information would create acceptance and trust and would avoid exaggerated expectations. Communication should be tailored to the target group. It should convey skepticism toward technology to younger people and introduce older people to the technology. Stakeholders wished for clarification on the fact that the technology can save time and does not take away expertise. Therefore, one should communicate that the outputs are basically suggestions, and the decision remains with the physician. Showrooms and practical examples should be used to convince people of the benefits and to create acceptance:

[...] I believe that it is very important for acceptance that you manage to make it clear that it is a system that can ultimately save time, which is then regained by the doctor for the patient.

Representative of quality assurance

We should certainly try to educate users and we do this by holding seminars, for example, where we remove the magic from AI and explain that it is machine learning. We also explain how it works and where the limits are. Because, as I always like to say, “there is no free lunch.

Researcher—AI in health care

“New professions” could also contribute to improvement. One expert explained the need for new professions to guarantee high data quality:

And I believe that if we don’t have new professional fields that can actually be called data secretaries or whatever, i.e. all those who actually ensure that the quality of the data is excellent, we will just have to make do with bad data and then also with bad results.

Representative of an ethics committee

Before the system becomes distributed, a “pretest” should be done in predefined settings. A system running in the background should serve to identify and check discrepancies in decisions between humans and the systems. Dissemination should begin in expert institutions to achieve a certain standard of quality and should always proceed from the most to the least competent users. During this process, systems should learn from mistakes:

That’s why there’s a phase of around six months where the system runs in parallel, in the background, where a comparison is made between what the machine would have decided and what the doctor would have done. A machine also learns from this so that it can then say when the system is introduced - I would never do an untested implementation.

Developer of AI-based CDSS

[...] at some point a state will be reached, a kind of steady state, in which it is very mature, [...] and then you can go further, but I think it is very important in the first few years to work together with the competent people and not to present it as a help that those who have resource problems or qualification problems can now do things with it that they would otherwise not be able to do.

Representative of quality assurance

In the context of AI-based CDSS, it should always be clear who the target users are. Therefore, the developer should clearly offer a “user definition.” Experts explained that systems should neither be used to “replace staff” nor to make up for a lack of specialist staff. Ideally, there should be teamwork or cooperation between the AI and users. AI-based CDSS should not undermine the expertise of physicians. While the systems should aim to support users, they should not overtake their task of decision-making:

Furthermore, I think it is important that we do not immediately say, yes, there is great dynamism, we can replace the missing specialist staff in a few years or save on staff now and eliminate an economic deficit.

Representative of quality assurance

Finally, two other possible improvements were seen in this category. First, software consulting firms should always be available as contact partners. Second, a self-fulfilling solution was seen over time. This was seen because, on the one hand, the use of good systems will automatically lead to acceptance and, on the other hand, because future generations are more open to AI.

## Discussion

### Principal Findings

The results of this study show that there is already a wide range of suggested improvements in the context of AI-based CDSS. These exist in 6 areas: “technology,” “data,” “user,” “studies,” “law,” and “general.” A structured overview of all identified solution strategies can be found in Multimedia Appendix 4. As in other studies on AI-based CDSS, improvements were found that address the technology itself, the users, and the context in which the systems are used [19-21]. Among others, the recommendations concerning the systems encompass the following aspects: a comprehensive developmental and evaluative process with the active engagement of users before the implementation; systematic training on an extensive, high-quality database; rigorous studies proving the system benefits; optimized ease of use and accessibility; customized systems tailored to specific settings, hospitals, and patient populations; and the incorporation of control mechanisms or the use of intelligent design and smart implementation to mitigate automation bias. On the user side, enhancements were suggested to the training program for (future) users. Training should include communication about the advantages as well as sensitization for critical thinking. Temporal valences of the users should be considered; systems should alleviate the burden on staff rather than augment their workload. Finally, regarding the context, experts suggested strategies such as increasing the level of digitalization of the health care system, especially hospitals; transparently communicating in public about the advantages and risks of AI; providing funding for research; clarifying open legal questions, for example, with regard to liability; and standardizing and consolidating various approval processes.

There are already some studies that investigate factors enabling or facilitating the use of AI-based CDSS. These stem from various fields such as antibiotic prescription in hospitals [19,20,22], rhythm management of atrial fibrillation [23], red blood cell transfusion [24], and aortic dissection [25]. For example, they focus on health professionals [19,23-25] or hospital managers [22]. Our study was not aimed at a special medical field or a single group of experts. Therefore, we were able to investigate indication-independent improvements from a broad range of different experts. This is especially important because several complex problems need interdisciplinary cooperation to be solved.

According to the experts interviewed in our study, a high-quality and user-friendly design of AI-based CDSS should be guaranteed. In this way, problems occurring during use could be prevented. One such problem is automation bias. To prevent this issue, systems should be designed with a well-thought-out design. This includes system outputs with fewer on-screen details, dynamic updates of the confidence levels of recommendations, and the provision of supportive information rather than orders [26]. Another example mentioned by experts and confirmed by the literature was transparency. Trust from physicians can be gained by providing as much transparency as possible [27]. An example not restricted to AI-based CDSS but relevant to digital solutions, in general, is the topic of usability. By integrating future users at every stage during the design process, ease of use can be enabled [28].

Both manufacturers and policy makers are in charge of guaranteeing a high quality of AI-based CDSS. While manufacturers should have an intrinsic motivation to develop high-quality systems, policy makers should set the framework conditions.

The EU first took the step of introducing the AI Act [29]. A prerequisite for the development of reliable AI-based CDSS is the availability of sufficient quality data. Therefore, experts stated that data should be collected in a standardized format and made available to researchers as well as private companies. One example of a large collection of physiological and clinical data is the PhysioNet platform, managed by members of the MIT Laboratory for Computational Physiology [30]. A German project funded by the EU is the SepsisDataNet.NRW [31]. One of the project’s goals is to create a sepsis-related biomarker database. From a legislative perspective, the German Bundestag adopted the Act on the Improved Use of Health Data (Health Data Use Act [Gesundheitsdatennutzungsgesetz]) [32]. At the European level, it is planned to create the “European Health Data Space,” an initiative to enable patients to easily control their electronic health data and to make this data available for research, innovation, and political decision-making [33].

Experts explained that the perception of users should be positively influenced. There is little experience with the new technology. In a survey conducted by Chen et al [34], only 27% of the physicians and medical students surveyed worldwide had used clinical AI. A total of 13% reported having good knowledge of AI. Furthermore, 77% expressed a high willingness to learn about the topic, and 78% agreed that training should be provided by hospitals or schools. Only 8% feared that physicians would be replaced by AI [34]. The low level of knowledge, together with a great openness [35,36] to the topic, provides an ideal basis for education. In this context, a special focus should lie on scrutinizing proposed decisions to prevent blind trust, automation bias, and alert fatigue.

Regarding studies, interviewees demanded more prospective randomized studies. This aligns with the existing literature. In 2022, a systematic review of randomized controlled trials (RCTs) of AI in clinical practice identified only 39 eligible studies and concluded with the need for more RCTs of AI-assisted tools integrated into clinical practice [37]. To enable such studies, national or international bodies should provide funding.

According to experts, regulation in the context of AI-based CDSS has not yet been fully elaborated. Questions regarding the trade-off between data protection and patient benefit, regarding the liability and the certification of AI-based CDSS, should be the subject of further discussion and research. For example, there is no uniform opinion between physicians and patients regarding liability for AI in health care [38].

The spectrum of general improvements proposed by experts was broad. Two topics should be particularly underlined. First, in Germany, where the interviews took part, many hospitals are still not sufficiently digitally equipped to enable the use of AI-based CDSS. Given the combination of the high costs of digitalization and the financially strained situation of most hospitals in Germany, the burden cannot be borne by the hospitals alone. Therefore, more funded initiatives, such as the SmartHospital.NRW initiative are required. The aims of this initiative are, on the one hand, to develop a process model that can be transferred to hospitals with different levels of digitalization and, on the other hand, to develop and test innovative, AI-based applications for real-world use-case scenarios [39]. Second, interviewees expressed a high need for education, not only for medical professionals but also for the public. Even if the attitude toward AI is generally positive, there are also some concerns or perceptions. For example, consumers fear the dehumanization of the clinician-patient relationship, see a threat to shared decision-making involving patients, assume low trustworthiness of AI advice, and express uncertainty around fairness and equity in treatment allocation [40]. These concerns should be taken seriously and addressed through open and transparent communication about the opportunities and limitations of AI.

### Implications

The suggested improvements should not be used without reflection because there is a risk that improvements will result in further problems. For example, experts suggested that information about the benefits of AI should be given. Thereby, on the one hand, acceptance could be improved, but on the other hand, there is a potential risk that users have too much confidence in the systems and are prone to blind trust, respectively automation bias. Another example is the critical attitude of users. Some experts stated that every decision proposed by the systems should be scrutinized. While, in general, this sounds comprehensible, it bears the risk that the systems might not lead to a significant reduction in workload and thus might become rejected by physicians.

Even if our research provides a variety of different improvement approaches, we did not search for an ideal match between existing problems and improvements. Even if, in some cases, the matching is obvious (eg, a lack of transparency could be reduced by providing decisive parameters), there are more complex interrelationships between some problems and solutions. For example, there is no direct solution for discrimination. Rather, various improvements should be considered here. An optimization of the system itself, the underlying database, or raising user awareness of the problem could be possible approaches. Therefore, further studies should be conducted to investigate such relationships.

The hierarchical framework from the study conducted by Kaiser et al [41] might serve as a starting point to identify the key implementation barriers and improvements that must be considered when introducing AI-based CDSS. According to this, the top level requires an effective implementation strategy (strategy level), the middle level should consider the capabilities and resources needed to successfully integrate an innovation, and finally the bottom level focuses on the implementation and change of processes and routines that are needed to accommodate the innovation.

### Recommendations

On the basis of the results of this study, some actionable, solution-oriented recommendations can be proposed that could be practically implemented by stakeholders (Table 2). These recommendations are not specific to the German health care system, as they address cross-cutting challenges, such as data quality, user behavior, regulatory complexity, and clinical validation—issues that arise independently of national health care structures. Their relevance extends to many countries facing similar demands in safely and effectively integrating AI-based CDSS into routine care.

**Table 2:** Solution-oriented recommendations based on this study.

Number	Recommendation	Problem	Solution
1	Postmarket monitoring	AI^a^-based CDSS^b^ should rely on the current state of medical knowledge. However, medical knowledge is subject to constant change, leading to concept and data drifts. Consequently, the original assumptions on which systems are based are no longer up to date, potentially reducing their accuracy or even their validity.	Regularly investigating the quality and reliability of AI-based systems might uncover drifts at an early stage. These verifications should not only be of a purely technical nature but also include actual use in care.^c^
2	Control mechanisms	A severe threat in using AI-based CDSS is automation bias.	Control mechanisms within the systems could be used to encourage users to think and avoid blindly implementing recommendations. One possible approach might be to provide system-based recommendations only after the assessment of the user.^d^
3	Comprehensive data pools	The quality and quantity of data are considered major problems. This may be particularly true for rare diseases.	Building supranational data pools can provide more data to train reliable AI-based CDSS. Common data standards, such as FHIR^e^, can support standardization. However, the special characteristics of health care systems and patients, as well as other potential factors arguing against generalization, should also be considered.^f^
4	User training	The perception of AI differs greatly. The spectrum ranges from exaggerated fear to unrealistic expectations. There are problems in both directions: either the systems are rejected, or users trust them blindly.	By integrating AI into the curricula of medical faculties, future users can engage with the technology in a more informed way. This could provide the skills to critically engage with AI-based CDSS. Thus, both automation bias and irrational fear can be met at an early stage.
5	Implementation of studies	User acceptance presents a possible hurdle for the introduction of AI-based CDSS in health care. While studies could be convincing because of the proof of benefit, the implementation of studies is associated with high costs.	Studies are essential to establish the evidence of AI-based CDSS. While current studies mostly focus on the accuracy of the algorithms (eg, recall and precision), future studies should also examine the actual benefits of their use in a clinical setting. Preferably, this should be done with RCT^g^ studies. However, as such studies are costly, they should be financed by national or supranational funds. Such an approach would enable universities and smaller independent institutes, in particular, to conduct research in this field.
6	Harmonization of approval procedures	The approval of medical systems is already associated with high effort, including outside Europe. This affects not only developers but also certifying bodies.	Future legislation (eg, the AI Act in Europe) should consider and harmonize existing regulations. This could significantly reduce workload and documentation burden. Possible areas include postmarket surveillance, risk assessment, safety and performance requirements, and technical documentation.

^a^AI: artificial intelligence.

^b^CDSS: clinical decision support systems.

^c^The European Union is introducing such postmarketing monitoring for high-risk products as part of the AI Act. The actual implementation is planned for February 2026.

^d^The actual design requires further research to prevent users from using “dummy inputs” to get recommendations. A cooperative process between the user and AI might be a promising approach.

^e^FHIR: Fast Healthcare Interoperability Resources.

^f^The European Union recently decided that such a data space (“the European Health Data Space”) will be created for the member states.

^g^RCT: randomized controlled trial.

While these recommendations appear well-founded and may offer valuable insights for optimizing AI-based CDSS and their integration into health care, it is important to emphasize that they are derived from a qualitative, exploratory approach. Therefore, they should be considered preliminary and require further investigation and validation. This will be addressed in the context of our overarching research project “KI@work” [14]. On the basis of the results of these expert interviews, supplemented by a literature review, focus groups with physicians and nursing staff, a quantitative questionnaire survey and workshops with experts, and well-founded recommendations for action are to be developed that are more binding in nature.

### Limitations

Some limitations of our research should be mentioned. First, we conducted a qualitative interview study. Therefore, the results are more exploratory than deterministic. In addition, the attitudes, values, and experiences of the interviewers may have impacted the final results. To minimize this risk, only a structuring and no further interpretation of the results was carried out. In addition, we have presented the methodological approach as transparently as possible and provided data (eg, appropriate citations in the paper and systematization of the statements in Multimedia Appendix 4) to substantiate the results. In our study, we focused on the German health care sector. Therefore, we only interviewed German experts. This might have an impact on the results for two reasons. First, some of the improvements identified could be specific to the German health care system. While, from our view, only a few improvements are affected by this, one should always scrutinize whether the individual identified improvements are transferable to other health care systems. Second, in the context of this publication, it was necessary to translate the individual statements into English. To avoid translation errors, we conducted quality checks of the citations within the consortium to guarantee that the initial meaning was not distorted.

Rather than providing an exhaustive list of all possible improvements, the objective of this study was to offer comprehensive perspectives. Therefore, it is unlikely that data saturation was fully achieved for each expert group. More specialized research should focus on specific groups, such as caregivers, patients, medical product consultants, experts in ethics and law, or manufacturers of AI-based CDSS, to identify conclusive lists of group-specific problems and improvements. Furthermore, the present findings should be further investigated and elaborated on in more detailed studies focusing on individual improvement categories or individual starting points for improvements, as well as in quantitative studies. A further practical step would be to systematically map implementation studies from the existing literature and examine them for successful improvement strategies.

Because AI-based CDSS are relatively new, there is no conclusive knowledge about underlying problems and barriers. Consequently, new problems and barriers could occur and require further solutions. Therefore, both the problems and barriers, as well as the improvements, should be continuously investigated and developed.

### Conclusions

There is evidence that AI-based CDSS have the potential to improve patient outcomes and optimize efficiency in health care. However, to fully realize these benefits and to guarantee safety for patients and a good user experience for medical staff, it is essential to urgently address the existing barriers and unresolved challenges. Possible strategies for improvement should address the systems, the users, and the environment in which they are used. Experts saw concrete points of contact in “technology,” “data,” “user,” “studies,” “law,” and “general.” These should serve as a starting point for further, detailed research.

## Appendix

Multimedia Appendix 1 COREQ (Consolidated Criteria for Reporting Qualitative Research) checklist.

Multimedia Appendix 2 Characteristics of moderators.

Multimedia Appendix 3 Interview guideline.

Multimedia Appendix 4 Systematization of the expert statements.

## Abbreviations

AI: artificial intelligence
CDSS: clinical decision support systems
COREQ: Consolidated Criteria for Reporting Qualitative Research
EU: European Union
RCT: randomized controlled trial

## Data Availability

With the exception of the full interview transcripts and the wording of all key statements, all relevant data can be found in the paper or in Multimedia Appendix 4. The complete transcripts, even in pseudonymized form, are not publicly available owing to data protection considerations. However, selected relevant passages are quoted verbatim in the manuscript to ensure transparency.
