# Supplementary material for: Improving AI-Based Clinical Decision Support Systems and Their Integration Into Care From the Perspective of Experts: Interview Study Among Different Stakeholders
Source: JMIR Med Inform. 2025 Jul 7;13:e69688. doi: 10.2196/69688 (PMC12280832; doi:10.2196/69688)
Supplement: Multimedia Appendix 2 [file medinform_v13i1e69688_app2.docx]

# Multimedia Appendix 2. Characteristics of moderators.

| Moderator | Sex | Back-ground | Experience | Professional position | Institution |
| --- | --- | --- | --- | --- | --- |
| GDG | m | (Health-)Economy | Has been involved in several research projects with qualitative components and has conducted several focus groups and interviews | Research associate | Institute for Healthcare Management and Research, University Duisburg-Essen, Germany |
| NB | f | (Health-)Economy and medical doctor | Has led several research projects with qualitative components and has conducted several focus groups and interviews | Head of the research team | Institute for Healthcare Management and Research, University Duisburg-Essen, Germany |
| PR | m | (Health-)Economy | First qualitative research project and moderation of interviews. Experience in conducting interviews through his previous work in market research. | Research associate | Institute for Healthcare Management and Research, University Duisburg-Essen, Germany |
