# Supplementary material for: Improving AI-Based Clinical Decision Support Systems and Their Integration Into Care From the Perspective of Experts: Interview Study Among Different Stakeholders
Source: JMIR Med Inform. 2025 Jul 7;13:e69688. doi: 10.2196/69688 (PMC12280832; doi:10.2196/69688)
Supplement: Multimedia Appendix 3 [file medinform_v13i1e69688_app3.docx]

# Multimedia Appendix 3. Interview guideline.

| **Introductions and opening question** | |
| --- | --- |
| **Open question** | **Concretization questions** |
| 1. "Where do you see the biggest problems in the context of AI-based CDSS?" | Concrete problems in the context of AI-based CDSS might be:   - Organizational and administrative problems - Legal problems - Institutional hurdles - Ethical problems - Technical problems |
| **General problems** | |
| **Open question** | **Concretization questions** |
| 1. "How do you rate the acceptance and trust (of users and patients) in AI-based CDSS in Germany?”   “How could acceptance be increased?” | X |
| **(Preclinical) development phase** | |
| **Open question** | **Concretization questions** |
| 1. "Where do you see problems in the development phase of AI-based CDSS?”   “How could these problems be countered?” | X |
| 1. “Do you see problems in Germany concerning the availability and quality of training data for AI-based CDSS? (Garbage-In, Garbage-Out)”   “How could these problems be countered?” | Problems relating to the formalized structure of the data:   - Structured data vs. unstructured data     Problems relating to data quality:   - Evaluation of data quality - What to do with incorrect or incomplete data?     Problems relating to data availability:   - Not enough data available? - Effect of too little training and test data? |
| 1. “Can there be problems with the generalizability of AI-based CDSS?”   “How could these problems be countered?” | Problems with generalizability?   - Limited (local or regional) data basis - Different indication-specific phenotypes - Generic character of the data sets - Risk of bias in the results |
| **Clinical use** | |
| **Open question** | **Concretization questions** |
| 1. "Where do you see problems with the clinical use of AI-based CDSS?”   “How could these problems be countered?” | X |
| 1. "Where do you see the biggest problems and barriers regarding the integration of AI-based CDSS?”   “How could these problems be countered?” | Possible problems with integration:   - Insufficient IT infrastructure - Lack of legal requirements - Unforeseeable effects |
| 1. "What do you think about the problem of false alerts or incorrect recommendations for action within the use of AI-based CDSS?”   “How could these problems be countered?” | X |
| 1. “Do you see a danger in the unchecked implementation of recommendations for action issued by an AI-based CDSS?”   “How could this problem be countered?” | X |
| 1. “Do you think that users’ lack of qualifications in dealing with AI-based CDSS could lead to problems?”   “How could this problem be countered?” | X |
| 1. “What challenges result from the self-learning further development of AI-based CDSS after their implementation?”   “How could these challenges be countered?” | X |
| End | |
| **Open question** | **Concretization questions** |
| 1. "Open points?"     "Further comments?" | X |
